# The m^6^A methylation landscape, molecular characterization and clinical relevance in prostate adenocarcinoma

**DOI:** 10.3389/fimmu.2023.1086907

**Published:** 2023-03-23

**Authors:** Chao Li, Dongyi Peng, Yu Gan, Lei Zhou, Weibin Hou, Bingzhi Wang, Peng Yuan, Wei Xiong, Long Wang

**Affiliations:** ^1^ Department of Urology, Third Xiangya Hospital, Central South University, Changsha, China; ^2^ Department of Urology, Xiangya Hospital, Central South University, Changsha, China

**Keywords:** prostate adenocarcinoma, RNA N6-methyladenosine, prognosis, molecular characterization, immune infiltration

## Abstract

**Background:**

Despite the recent progress of therapeutic strategies in treating prostate cancer (PCa), the majority of patients still eventually relapse, experiencing dismal outcomes. Therefore, it is of utmost importance to identify novel viable targets to increase the effectiveness of treatment. The present study aimed to investigate the potential relationship between N6-methyladenosine (m6A) RNA modification and PCa development and determine its clinical relevance.

**Methods:**

Through systematic analysis of the TCGA database and other datasets, we analyzed the gene expression correlation and mutation profiles of m6A-related genes between PCa and normal tissues. Patient samples were divided into high- and low-risk groups based on the results of Least Absolute Shrinkage and Selection Operator (LASSO) Cox analysis. Subsequently, differences in biological processes and genomic characteristics of the two risk groups were determined, followed by functional enrichment analysis and gene set enrichment (GSEA) analysis. Next, we constructed the protein-protein interaction (PPI) network of differentially expressed genes between patients in high- and low-risk groups, along with the mRNA-miRNA-lncRNA network. The correlation analysis of tumor-infiltrating immune cells was further conducted to reveal the differences in immune characteristics between the two groups.

**Results:**

A variety of m6A-related genes were identified to be differentially expressed in PCa tissues as compared with normal tissues. In addition, the PPI network contained 278 interaction relationships and 34 m6A-related genes, and the mRNA-miRNA-lncRNA network contained 17 relationships, including 91 miRNAs. Finally, the immune characteristics analysis showed that compared with the low-risk group, the levels of M1 and M2 macrophages in the high-risk group significantly increased, while the levels of mast cells resting and T cells CD4 memory resting significantly decreased.

**Conclusions:**

This study provides novel findings that can further the understanding of the role of m6A methylation during the progression of PCa, which may facilitate the invention of targeted therapeutic drugs.

## Introduction

According to the statistics of the American Cancer Society, prostate cancer (PCa) is the second leading cause of cancer-related death in men in the United States, with an estimated 288,300 new cases and 34,700 deaths per year, accounting for 28.5% and 10.8% of all cancers, respectively (1). With the substantial increase in the aging population in China, the incidence of PCa has also increased year by year, and PCa has become the most common urogenital tumor in elderly men (2). Despite recent advances in surgical and drug treatments, the mortality rates of patients with recurrent or metastatic PCa remain close to 100% (1). Therefore, in-depth study of molecular markers related to treatment and prognosis of PCa and searching for more effective therapeutic targets are of significant importance for the clinical benefit of PCa patients.

To date, more than 150 RNA post-transcriptional modifications have been identified in eukaryotes (3). N6-methyladenosine (m^6^A) is the most common RNA modification in mammalian cells that has important roles in different biological processes (4, 5). Abnormalities in regulatory mechanisms of m^6^A have been identified as involved in a variety of human diseases including cancer (6). m^6^A, as the methylation at the sixth N position of adenylate in RNA, is the most common modification of RNA in eukaryotes, accounting for about 80% of RNA methylation modifications, and each mRNA contains 3 to 5 m^6^A residues on average (3). This process is dynamically and reversibly regulated by methyl transfer-related proteins (METTL3, METTL14, and WTAP, etc.) and demethylases (FTO, ALKBH3, and ALKBH5, etc.), and affects various steps of mRNA metabolism reader, including mRNA processing, nuclear export, translation and degradation, by binding to the m^6^A (7). Several studies have established the model for m^6^A risk-related prognosis to evaluate the treatment effect and prognosis of metastatic PCa, finding that in patients with metastatic PCa, a higher m^6^A risk score indicates a worse prognosis, which is significantly associated with biological functions such as DNA mismatch repair. Therefore, patients with high m^6^A risk scores may be a more suitable population for DNA repair-targeted drug therapy (8, 9). In addition, several studies have reported the potential tumor-promoting or tumor-suppressing effects of m^6^A methylation-related factors such as METTL3, METTL14 and FTO in PCa (10–14). However, there is still a lack of integrative analysis of the expression of m^6^A RNA methylation regulator, clinicopathological features, malignant progression, and prognosis in PCa.

In this study, we used published sequencing data to investigate the possible role of m^6^A methylation in the progression of PCa, and to establish relevant clinical prediction model to analyze the predictive power of prognosis in PCa.

## Materials and methods

### Data acquirement and processing

The gene expression data of gene sequencing of patients with prostate adenocarcinoma (PRAD) was downloaded from the TCGA GDC (https://portal.gdc.cancer.gov/). The clinical characteristics of the corresponding patients, including age, gender, and survival prognosis, were also downloaded. After deleting the PRAD patients with missing clinical information, 481 tumor tissues and 51 normal tissues were ultimately included in the analysis. The somatic mutation data of PRAD patients were downloaded and maftools package of R software was used to visualize the somatic mutation (15). The tumor mutation burden (TMB) of each patient was collected. Besides, datasets including GSE46602 and GSE69223 were downloaded from the Gene Expression Omnibus (GEO) database (https://www.ncbi.nlm.nih.gov/geo/) (16, 17). Moreover, GSE46602 contains 36 tumor tissues and 14 normal tissues, and GSE69223 contains 15 tumor tissues and 15 normal tissues. Both datasets came from the GPL570 sequencing platform, where the species origin was Homo sapiens.

### Construction of a risk model for PCa

To analyze the expression of m^6^A-related genes in PRAD, we first analyzed the differential expression and gene expression correlation of m^6^A-related genes in PRAD and normal tissues. The risk genes associated with PCa prognosis were obtained through univariate cox regression analysis of the expression and survival of PRAD patients from TCGA. The risk genes associated with PCa prognosis were subsequently incorporated into the model, and the Least Absolute Shrinkage and Selection Operator (LASSO) was used to reduce the data dimensionality and obtain prognostic-related signature genes. The normalized values of expression of each gene were weighted by the penalty coefficient by LASSO Cox analysis, a risk score formula was established, and the patients were divided into high-risk group and low-risk group according to median value of the risk score, as follows:


$$\text{riskScore }=\;\sum_{\text{i}}\text{Coefficient }\left({\text{risk gene}}_{\text{i}}\right)*\text{mRNA Expression }\left({\text{risk gene}}_{\text{i}}\right)$$

### Differentially expressed genes analysis

To analyze the effect of risk score on DEGs analysis of PRAD, the R package “DESeq2” was used to perform DEGs analysis on samples in high-risk and low-risk groups of the dataset from TCGA-PRAD to screen for significant differential genes (18). The absolute value of log2 fold change (logFC) > 1.5 and Padj< 0.05 were set as the thresholds of differential genes. Genes with logFC > 1.5 and Padj< 0.05 were up-regulated DEGs, and genes with logFC< -1.5 and Padj< 0.05 were down-regulated DEGs (19).

### Genomic characteristics and biological characteristics of patients in high-risk group and low-risk group

Following the development of tumor genomics, the Mutation Annotation Format (MAF) has become widely accepted and used to store detected somatic variants. In order to evaluate the variation of gene copy number variation in risk-grouping, the GISTIC2.0 in the Genepattern (https://cloud.genepattern.org/) analysis platform was used to analyze the copy number variation in the risk groups of TCGA database (20).

In this study, the MSIpred method was used to analyze the relationship between risk-grouping and TMB or microsatellite instability (MSI), respectively (21). In addition, in order to investigate the variation of biological process of samples in high-risk group compared with that in low-risk group, we performed gene set variation analysis using the R package “GSVA” based on the gene expression profiling dataset of PRAD patients from TCGA (22).

The reference gene set “h.all.v7.4.symbols.gmt” was downloaded from the MSigDB database to calculate the enrichment score of each sample in each pathway in the dataset (23), and evaluate the relationship between the enrichment score and the risk score. *P*< 0.05 was considered statistically significant.

### Functional enrichment analysis and gene set enrichment analysis

GO analysis is a method commonly used for large-scale functional enrichment studies, including biological process (BP), molecular function (MF) and cellular component (CC) (24). KEGG is a widely used database for storing data about genomes, biological pathways, diseases, and drugs (25). GO annotation analysis and KEGG pathway enrichment analysis of differentially expressed genes were performed using the clusterProfiler package of R and a cutoff value of FDR< 0.05 was considered statistically significant (26).

To investigate differences in biological processes between two groups, based on the gene expression profiling dataset of PRAD patients, gene set enrichment analysis was performed using GSEA, which is a computational method to analyze the potential existence of significant differences in a specific gene set between two biological states (27). Also, GSEA is often used to estimate changes in pathway and biological process activity in samples of expression dataset. The “c2.cp.kegg.v7.4.symbols.gmt” gene set and the “c5.go.v7.2.symbols.gmt” gene set were downloaded from the MSigDB database for GSEA analysis. *P*< 0.05 was considered statistically significant.

### Identification and correlation analysis of tumor infiltrating immune cells

CIBERSORT is an algorithm that deconvolves the expression matrix of immune cell subtypes based on the principle of linear support vector regression, which utilizes RNA-Seq data to estimate the abundance of immune cells in tissues (28). The CIBERSORT in R software was used to estimate the abundance of 22 kinds of immune cells in high-risk and low-risk groups in the dataset, and boxplots were performed to visualize the immune cell composition of disease samples and normal samples. The Wilcoxon test calculated differences in the proportion of immune cells between disease samples and normal samples, and *P*< 0.05 was considered statistically significant. The dataset on the interaction of PRAD cell lines with drugs was obtained from the GDSC database (29), and the R package oncoPredict was used for drug sensitivity analysis of the expression data of patients in the high-risk group and the low-risk group from TCGA-PRAD so as to compare the sensitivity differences in anti-tumor drugs between patients in high-risk group and low-risk group (30).

### Construction of protein-protein interaction network and key gene-miRNA network

The PPI network includes interactions of individual protein with each other that participate in all aspects of life processes such as biological signal transmission, gene expression regulation, energy and material metabolism, and cell cycle regulation. Therefore, systematic analysis of the interaction of a large number of proteins in biological systems is useful for elucidating the working principle of proteins in biological systems, understanding the mechanism of biological signals and energy metabolism under special physiological conditions such as diseases, as well as the functional connections between proteins.

The STRING database is used for searching for interactions between known protein and predicted protein (31). In this study, we used the STRING database and selected genes with a combined score > 400 to construct a protein-protein interaction network related to DEGs. Besides, Cytoscape (v3.7.2) was used to visualize the PPI network model. Genes in the PPI network were functionally annotated using clueGO (32, 33).

In order to analyze the relationship between key genes and miRNAs in the post-transcriptional stage, miRNAs related to differentially expressed genes from the miRNet database were obtained to construct an mRNA-miRNA regulatory network (34). The mRNA-miRNA regulatory network was visualized using Cytoscape software. lncRNA is a class of RNA molecules with transcripts longer than 200 nt, which are generally considered to not encode proteins, but participate in the regulation of protein-coding genes in the form of RNA in epigenetic regulation, transcriptional regulation and post-transcriptional regulation (35).

To analyze the relationship among DEGs and miRNAs and lncRNAs in the post-transcriptional stage, we obtained miRNAs and lncRNAs related to DEGs from the miRNet database to construct an mRNA-miRNA-lncRNA regulatory network (34), which was visualized by the Cytoscape software.

### Construction of clinical prediction model based on risk model

To demonstrate the individualized assessment of prognosis of patients by risk scores combined with clinicopathologic characteristics, univariate and multivariate Cox analyses were subsequently performed to analyze the predictive power of risk scores combined with clinicopathologic characteristics of patients for overall survival (OS). Subsequently, the risk score model with clinicopathologic characteristics was selected to construct a clinical predictive nomogram. To quantify discriminative performance, a calibration curve was generated to assess the performance of the nomogram by comparing the predicted value of the nomogram with the observed actual survival.

### Cell culture

Human prostate normal cell line RWPE-1, PCa cell line 22Rv1 and PC3 were purchased from American type culture collection (ATCC). All cells were cultured in RPMI-1640 cell culture medium containing 10% FBS in a 5% CO_2_ humidified atmosphere at 37°C. When used in experiments, these cell lines were cultured within 20 passages, and regular routine testing was employed to confirm them as negative for mycoplasma.

### Real-time-qPCR analysis

In order to detect the mRNA levels of each m^6^A-related factor, total RNA was extracted from cells using the RNAsimple Total RNA Kit (TIANGEN), after which the obtained RNA was reverse transcribed into cDNA using the RevertAid First Strand cDNA Synthesis Kit (ThermoFisher). Each cDNA sample was amplified using SuperReal PreMix Plus SYBR Green Supermix (TIANGEN) in the LightCycler 480 Real-Time PCR System (Roche) following the manufacturer’s instructions. Primers used for RT-qPCR analysis are shown in **Supplementary Table 1**. Relative RNA levels were calculated using the 2^-ΔΔCt^ method, and normalized to β-actin as an internal control.

### Western blot

To denature proteins, cell lysates were added to 5× loading buffer (Beijing TDY Biotech) and heated to 95°C for 5 min. Protein samples were separated by SDS-PAGE electrophoresis, transferred semi-dry onto NC membranes (Millipore), and blocked in Tris-buffered saline-Tween 20 (TBST) containing 5% nonfat milk for 30 min, after which the immunoblotting was performed by incubating with the primary antibody for 10 min at room temperature, and then overnight at 4°C. After being subjected to 5 washes, the membranes were incubated with goat anti-mouse/rabbit IgG (H+L)-HRP secondary antibody (Beijing TDY Biotech, 1:10000 dilution) for 40 min and were subsequently exposed to light using western ECL Substrate (Millipore). The relative expression levels of each protein were assessed using ImageJ software. Primary antibodies used in this study are listed in **Supplementary Table 2**.

### Statistical analysis

All data processing and analysis were performed by R software (version 4.1.1). The student’s t-test was used to estimate the statistical significance of normally distributed variables for the comparison of measurement data between two groups. The Wilcoxon rank-sum test was used to calculate the statistical significance of non-normally distributed variables between two groups. The Chi-square test or Fisher’s exact test was used to compare and analyze the statistical significance of categorical data between two groups. Correlation coefficients between different genes were calculated by Pearson correlation analysis. The Kaplan-Meier survival curve was used to show the difference in survival, and the log-rank test was used to evaluate the significant difference in survival between the two groups. All statistical P values were two-sided, and *P*< 0.05 was considered statistically significant.

## Results

### Expression and mutation of m^6^A-related genes in PRAD patients

**Figure 1:**
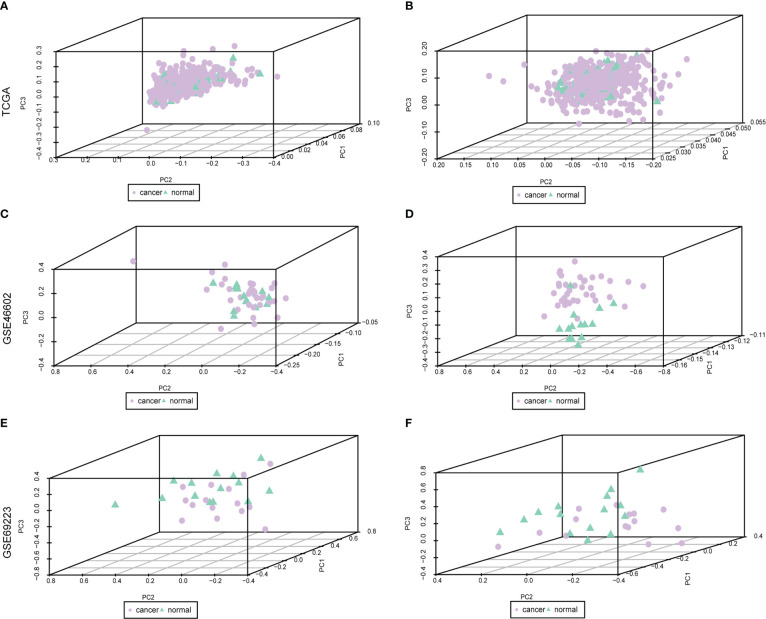
Dataset on PRAD after correction. Purple nodes indicate tumor samples, and green nodes indicate normal samples. **(A, C, E)** are the data before correction, and **(B, D, F)** are the data after correction.

The baseline data of patients with PRAD are shown in **Supplementary Table 3**. To analyze the expression levels of m^6^A-related genes in PRAD patients, we analyzed genomic mutations and mRNA expression, respectively. First, a comprehensive analysis of expression profiles in PCa tissues and normal tissues from TCGA data and GEO data was performed with de-batch effects (**Figure 1**). Principal Component Analysis (PCA) showed significant differences in m^6^A-related gene signatures between PRAD tissues and normal tissues.

**Figure 2:**
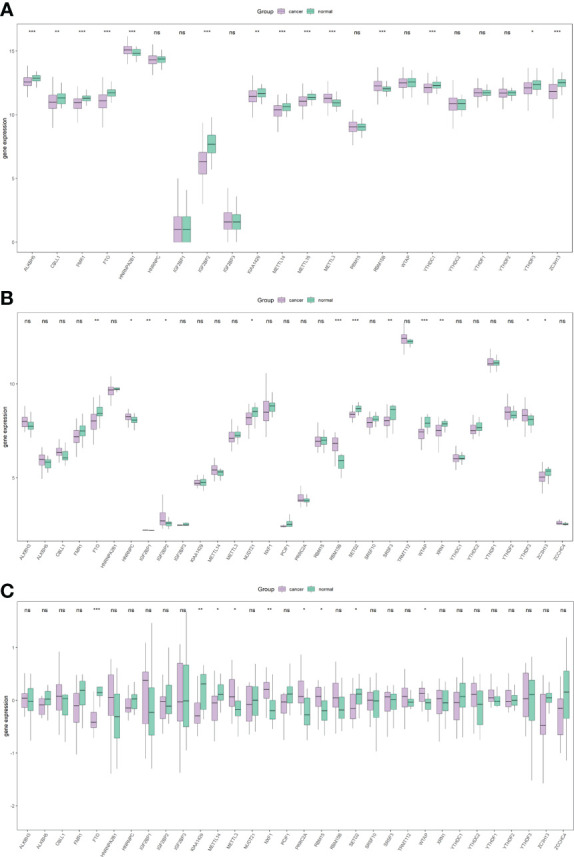
Overall expression of m^6^A-related genes in PRAD patients. Purple indicates the tumor sample, and green indicates the normal sample. Three images indicate TCGA **(A)**, GSE46602 **(B)**, GSE69223 **(C)**. *P < 0.05, **P < 0.01, ***P < 0.001, ns, not significant.

Subsequently, the differential analysis showed that a variety of m^6^A-related genes were significantly differentially expressed between PCa tissues and normal tissues, including FTO, METTL14, METTL16, ZC3H13, YTHDC1, YTHDF3, RBM15B, etc. (**Figure 2**).

**Figure 3:**
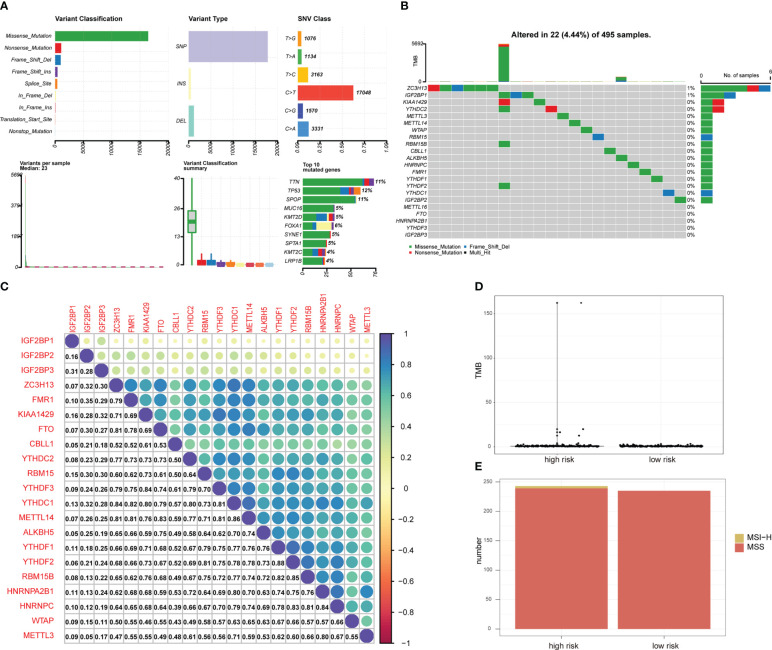
Mutation status of m^6^A-related genes in PRAD patients. **(A)** Summary of PRAD patients mutation data from TCGA. **(B)** Mutation map of m^6^A-related genes in PRAD patients from TCGA. Samples are ordered according to somatic nonsynonymous mutational burden and genes are ordered by mutation frequency, with various colors indicating different mutation types. Subsection above legend shows mutational burden. **(C)** The expression level correlation of m^6^A-related genes in the gene expression profile of PRAD patients from TCGA. The numbers in the figure and the annotation bar on the right indicate the magnitude of the correlation. **(D)** Differences in TMB between PRAD patients in high-risk group and low-risk group. **(E)** Differences in MSI status between PRAD patients in high-risk group and low-risk group.

Mutation analysis showed that most of the mutations were missense mutations, and most of the mutation types were SNPs (**Figure 3A**). There were 22 patients with PRAD and single nucleotide mutations in m^6^A-related genes, among which the ZC3HI3 had the highest mutation rate (**Figure 3B**). The correlation analysis of the heat map showed a positive correlation of m^6^A-related genes in PRAD tissues (**Figure 3C**).

The total number of mutations was obtained to calculate the TMB of the high-risk group of PRAD patients and low-risk PRAD patients. TMB was higher in PRAD patients in the high-risk group (**Figure 3D**), suggesting that PRAD patients in the high-risk group may be more likely to respond to immunotherapy. MSI is also an important treatment for predicting the effect of immunotherapy. Thus, we predicted the status distribution of MSI-H and MSI of PRAD patients in the high-risk group and low-risk group based on mutation data (**Figure 3E**). Our results showed that patients with MSI-H were all PRAD patients in the high-risk group and that MSI-H samples may be more sensitive to immunotherapy and more benefit from immunotherapeutic drugs.

### Construction of risk model and prognostic analysis

**Figure 4:**
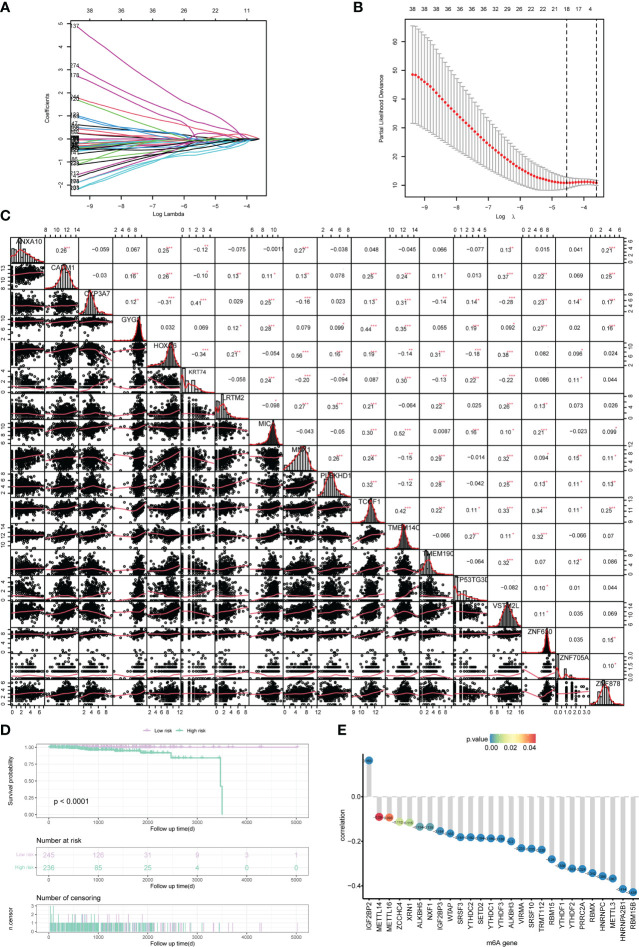
Construction of the risk scoring model. **(A, B)** LASSO Cox analysis identified 18 signature genes most associated with OS in the dataset of PRAD patients from TCGA. **(C)** Expression correlation analysis of signature genes in PRAD. **(D)** Kaplan-Meier curve assessed the effect of risk score on overall survival in PRAD patients, with patients with low risk in purple and patients with high risk in green. **(E)** The correlation analysis of m^6^A-related genes and risk scores. The horizontal axis shows m^6^A-related genes, the vertical axis shows the size of correlation, and the node color indicates the significance level. *P < 0.05, **P < 0.01, *** P < 0.001.

In order to analyze the impact of genes on the prognosis of PRAD patient, 278 risk genes associated with PCa prognosis were identified by univariate cox regression analysis, and enrolled in LASSO-Cox analysis to select and obtain 18 genes with the best prognostic value (**Figures 4A, B**). Subsequently, the correlation among the expression levels of these genes was analyzed, which showed that the signature genes were broadly represented (**Figure 4C**). At the same time, based on penalty coefficients of important signature genes calculated by LASSO-Cox analysis, the gene expression was multiplied by the corresponding coefficients and added to establish a risk score. Besides, the final risk score of each sample was calculated. Next, patients were divided into high-risk group and low-risk group based on the mean value of PRAD patients’ risk scores. Kaplan-Meier analysis showed that patients in high-risk group had relatively poor OS (Log-rank *P*< 0.0001, **Figure 4D**). Moreover, a significant correlation was found between the expression levels of m^6^A-related genes and the risk score of patients (**Figure 4E**).

**Figure 5:**
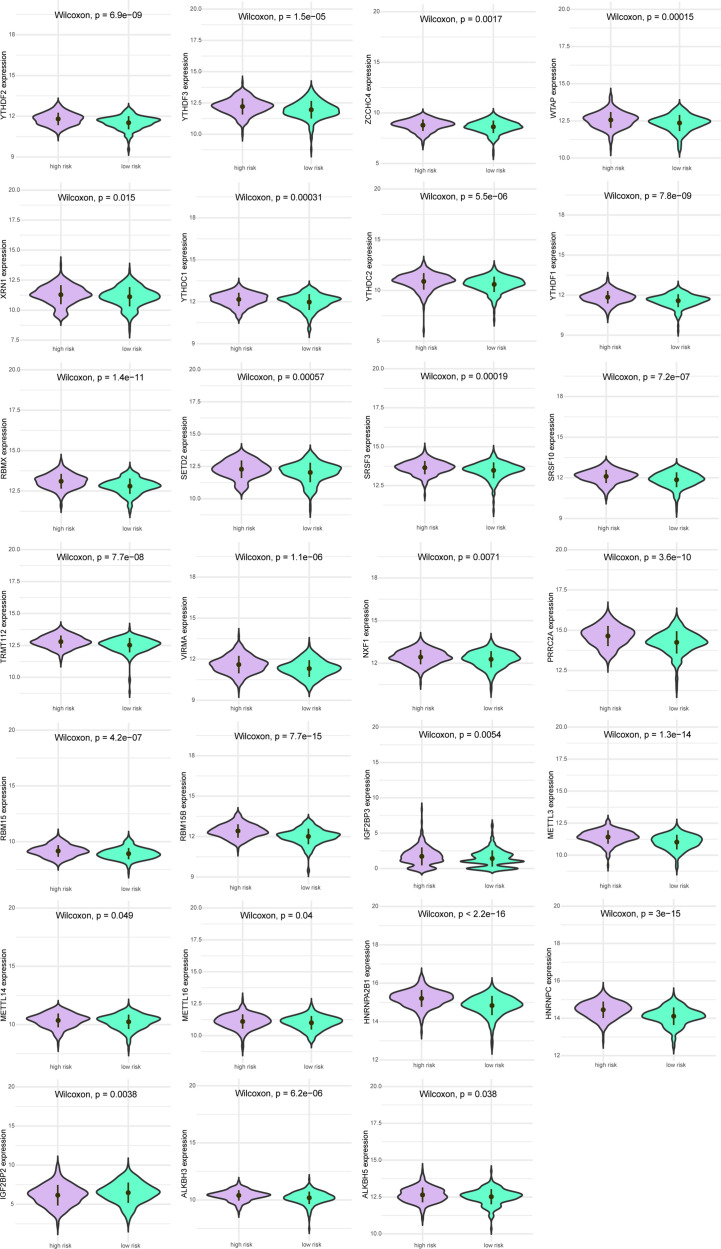
Expression levels of m^6^A gene between patients in a high-risk group and low-risk group. Purple indicates patients with low-risk, and green indicates patients with high-risk.

Next, we analyzed the differences in m^6^A-related gene expression levels of patients between the high-risk group and low-risk group, finding 27 m^6^A-related genes with significantly differential expression between patients in high-risk group and low-risk group (all *P*<0.05, **Figure 5**).

### Differences in biological processes and genomic characteristics of risk-groups

**Figure 6:**
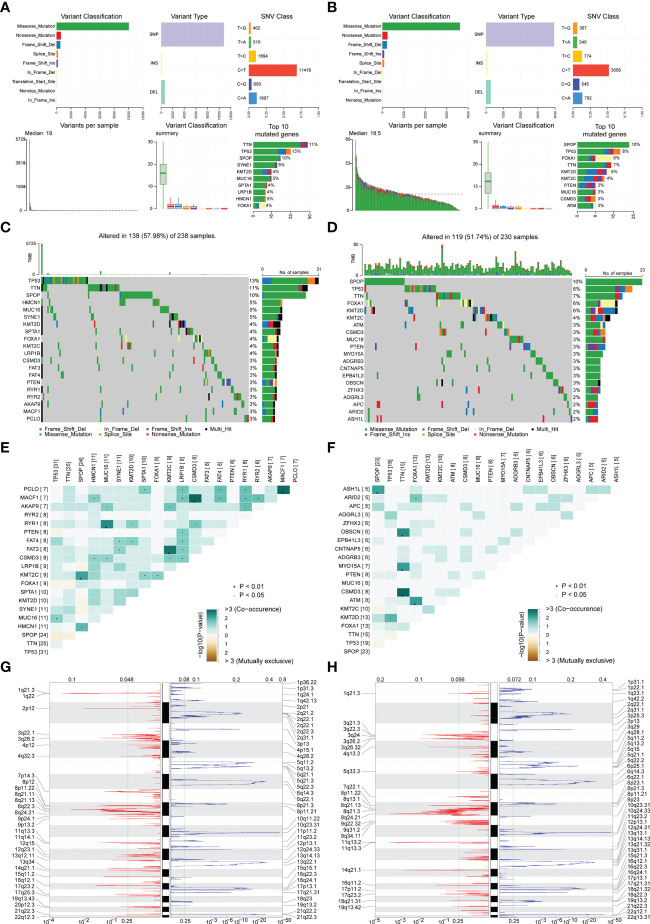
Correlation analysis of risk scores and genomic characteristics. **(A, B)** Summary data on mutation for patients with low-risk and patients with high-risk. **(C, D)** Statistics of top 20 mutant genes in patients with high-risk and patients with low-risk. Samples are ordered according to somatic nonsynonymous mutational burden and genes are ordered by mutation frequency, with various colors indicating different mutation types. The subsection above the legend shows mutational burden. **(E, F)** Demonstration of synergy and mutational relationships between mutated genes in patients with high-risk and patients with low-risk. **(G, H)** Identified genes with significant amplifications and deletions in patients with high-risk and patients with low-risk. Q-value and change score of GISTIC2.0 (x-axis) versus genomic location (y-axis). Dashed lines indicated centromeres. The green line represents the 0.25 Q-value cut-off point for determining significance. *P < 0.05.

The mutation types of mutated genes in PRAD patients in the high-risk group and low-risk group were analyzed, and more gene mutations were found in PRAD patients in the high-risk group (**Figures 6A, B**). Subsequently, we analyzed the high-frequency mutation genes of patients in the two groups, finding that the gene with the highest mutation frequency of patients in the high-risk group was TP53 (**Figure 6C**), while the gene with the highest mutation frequency among patients in the low-risk group was SPOP (**Figure 6D**). The relationship between mutated genes of patients in the two groups was compared, showing significant co-mutation between MACF1 and PCLO in PRAD patients in the high-risk group (**Figure 6E**), and significant co-mutation between SPOP and ASH1L in PRAD patients in the low-risk group (**Figure 6F**).

Finally, GISTIC 2.0 was used to identify genes with significant amplification or deletion in the copy number variation data of patients in two groups, respectively. The results showed more gene copy number amplifications on chromosomes 2, 12, 13, 20, and 21 in PRAD patients in the high-risk group (**Figures 6G, H**).

**Figure 7:**
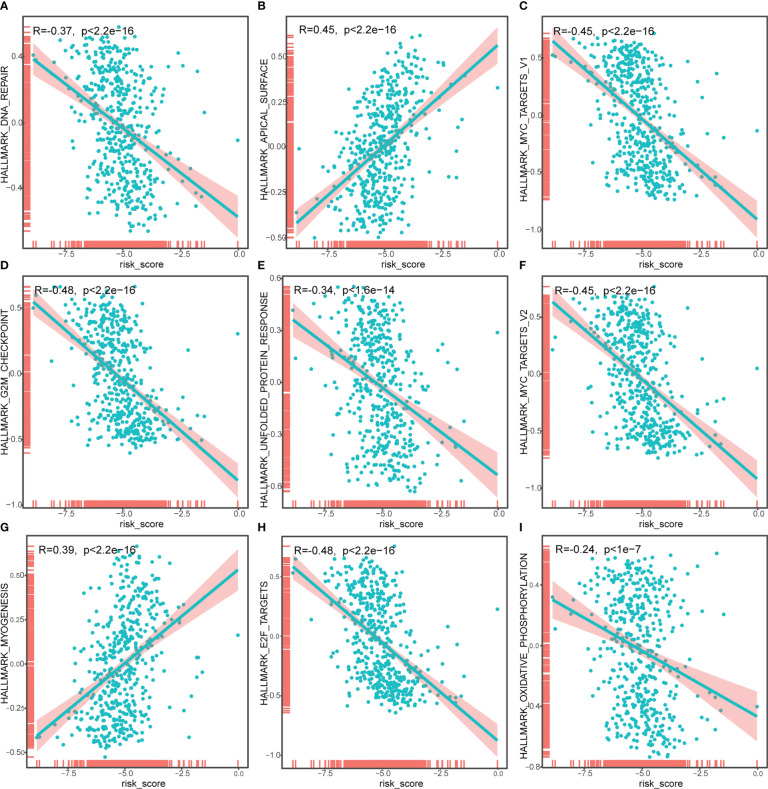
Correlation analysis of risk score and Hallmark_DNA_repair **(A)**, Hallmark_APICAL_surface **(B)**, Hallmark_myc_targets_v1 **(C)**, Hallmark_G2M_checkpoint **(D)**, Hallmark_unfolded_protein_response **(E)**, Hallmark_myc_targets_v2 **(F)**, Hallmark_myogenesis **(G)**, Hallmark_E2F_targets **(H)**, Hallmark_oxidative_phosphorylation **(I)**. The horizontal axis represents the risk score, and the vertical axis represents the enrichment score of the hallmark.

To identify the underlying biological features of the different risk models, we calculated the correlation between the enrichment score and the risk score at the hallmark for each sample, and the results showed that the risk score had a significant negative association with DNA repair, MYC targets V1, G2M checkpoint, unfolded protein response, MYC targets v2, E2F targets and oxidative phosphorylation, and significant positive association with an apical surface and myogenesis (all *P*<0.05, **Figure 7**).

### Difference analysis between high-risk group and low-risk group

**Figure 8:**
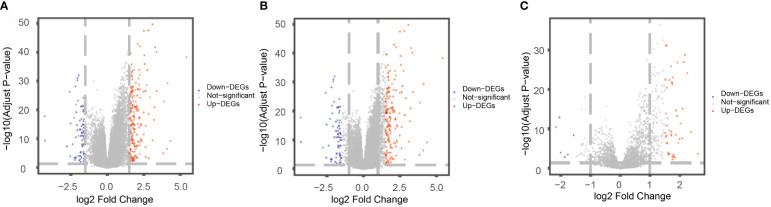
Differentially expressed mRNAs **(A)**, miRNAs **(B)**, lncRNAs **(C)** between patients in a high-risk group and a low-risk group. The horizontal axis was logFC; the vertical axis was -log10 (Adjust P-value). Red nodes represent up-regulated differentially expressed genes, blue nodes represent down-regulated differentially expressed genes, and gray nodes represent genes that were not significantly differentially expressed.

As the level of risk has a significant impact on the survival rate of patients, we conducted a differential analysis on the gene expression of patients in the high-risk group and the low-risk group, taking the genes with Padj< 0.01 and |logFC|> 1.5 as the differentially expressed genes. We identified 284 differentially expressed genes, including 207 up-regulated genes and 77 down-regulated genes (**Figure 8A**). At the same time, the differentially expressed genes were divided into differentially expressed mRNAs and differentially expressed lncRNAs. There were 164 up-regulated miRNAs and 71 down-regulated miRNAs (**Figure 8B**) identified, and 43 up-regulated lncRNAs and 6 down-regulated lncRNAs (**Figure 8C**).

**Figure 9:**
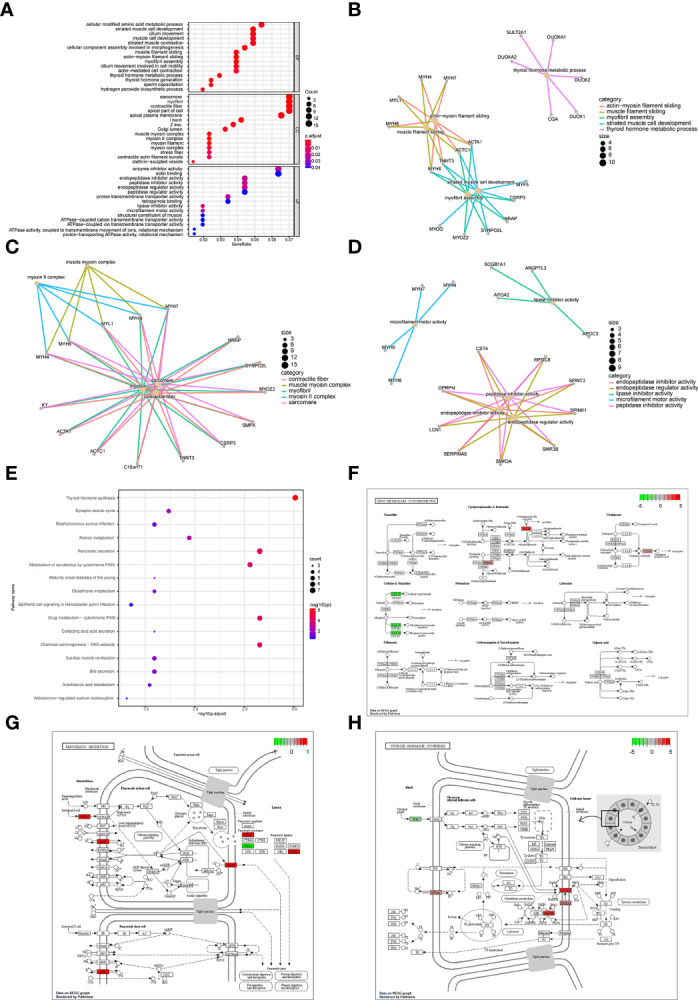
Enrichment analysis of differentially expressed genes between patients in a high-risk group and low-risk group. **(A)** GO functional enrichment analysis, the vertical axis is gene ratio, the horizontal axis is GO terms, the node color indicates -log10 (p value), and the node size indicates the number of genes contained in the current GO Term. **(B)** The first 5 items of BP are listed, the node’s size indicates the number of genes contained in the current GO Term, and the different colors indicate different GO Term. **(C)** The first 5 items of CC are listed, the node size indicates the number of genes contained in the current GO Term, and the different colors indicate different GO Term. **(D)** The first 5 items of MF are listed, the node size indicates the number of genes contained in the current GO Term, and the different colors indicate different GO Term. **(E)** KEGG pathway enrichment analysis, the horizontal axis was -log10 (p value), the vertical axis is the Pathway name, the node size indicates the number of genes enriched in the pathway, and the node color indicated -log10 (p value). **(F)** KEGG pathway with significant enrichment. hsa00982: Drug metabolism - cytochrome P450. **(G)** KEGG pathway with significant enrichment, hsa04972: Pancreatic secretion. **(H)** KEGG pathway with significant enrichment, hsa04918: Thyroid hormone synthesis.

Subsequently, we analyzed the impact of differentially expressed mRNAs between the high-risk group and low-risk group on biologically relevant functions of patients. First, GO functional annotation was performed on the differentially expressed genes (**Figure 9A**; **Supplementary Table 4**), revealing that these differentially expressed genes were mainly enriched in biological processes including muscle filament sliding, actin-myosin filament sliding, striated muscle cell development, myofibril assembly, thyroid hormone metabolic process, cellular component assembly involved in morphogenesis and thyroid hormone generation (**Figure 9B**); in cellular components including sarcomere, myofibril, contractile fiber, muscle myosin complex, and myosin II complex (**Figure 9C**), and in molecular functions including lipase inhibitor activity, endopeptidase Inhibitor activity, peptidase inhibitor activity, microfilament motor activity, endopeptidase regulator activity, enzyme inhibitor activity (**Figure 9D**). At the same time, these differentially expressed genes were enriched in KEGG pathways such as Thyroid hormone synthesis, Chemical carcinogenesis-DNA adducts, Pancreatic secretion, Drug metabolism-cytochrome P450 (**Figure 9E**; **Supplementary Table 5**). The enrichment of the expression levels of differentially expressed genes in pathways hsa00982, hsa04918, and hsa04972 is shown in detail in **Figures 9F–H**.

**Figure 10:**
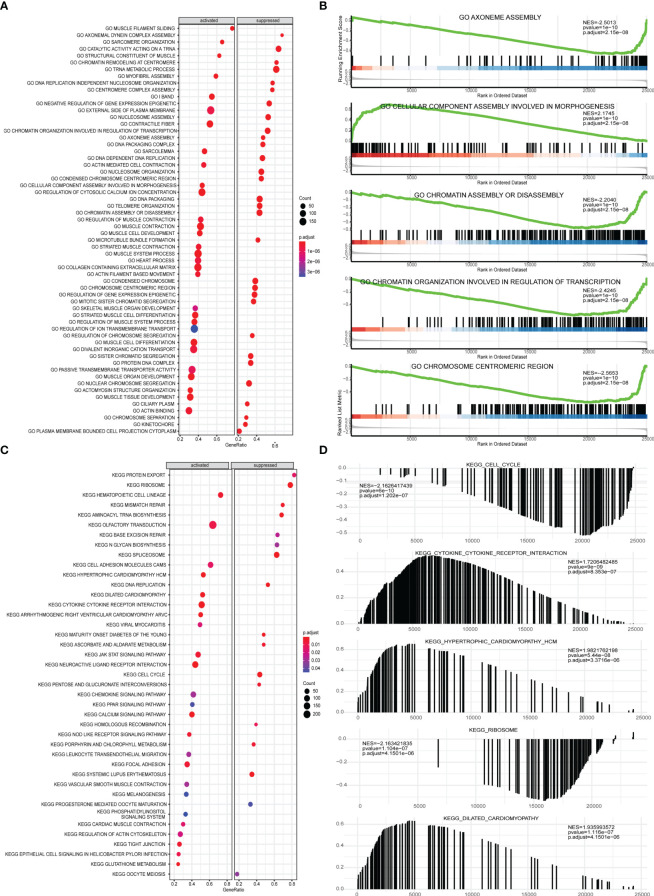
GSEA analysis of high-risk group and low-risk group. **(A)** GSEA-GO analysis of a dataset of PRAD patients from TCGA, the horizontal axis is the gene ratio, the vertical axis is the GO terms, and the color represents -log10 (p value). **(B)** The first 5 items of the GSEA-GO analysis of the entire dataset of PRAD patients from TCGA are shown. **(C)** GSEA-KEGG analysis of dataset of PRAD patients from TCGA, the horizontal axis is the gene ratio, the vertical axis is the GO terms, the node size represents the number of genes enriched in GO terms, and the node color represents log10 (p value). **(D)** The first 5 items of the GSEA-KEGG analysis of dataset of PRAD patients from TCGA.

Next, GSEA was performed on all gene expressions between the high-risk group and the low-risk group, showing significant differences in the following biological processes between groups (**Supplementary Table 6**). Among them, biological processes such as centromere complex assembly, mitotic sister chromatid segregation, DNA replication independent nucleosome organization, kinetochore, and axoneme assembly were inhibited, while biological processes such as myofibril assembly, contractile fiber, muscle filament sliding, sarcomere organization, and structural constituent of muscle were activated (**Figures 10A, B**). Meanwhile, it was found that pathways involved in hypertrophic cardiomyopathy, dilated cardiomyopathy, arrhythmogenic right ventricular cardiomyopathy, glutathione metabolism, cytokine-cytokine receptor interaction were activated, while pathways involved in cell cycle, maturity onset diabetes of the young, aminoacyl tRNA biosynthesis, mismatch repair, ribosome were inhibited (**Figures 10C, D**).

### PPI network of differentially expressed genes between patients in high-risk group and low-risk group

**Figure 11:**
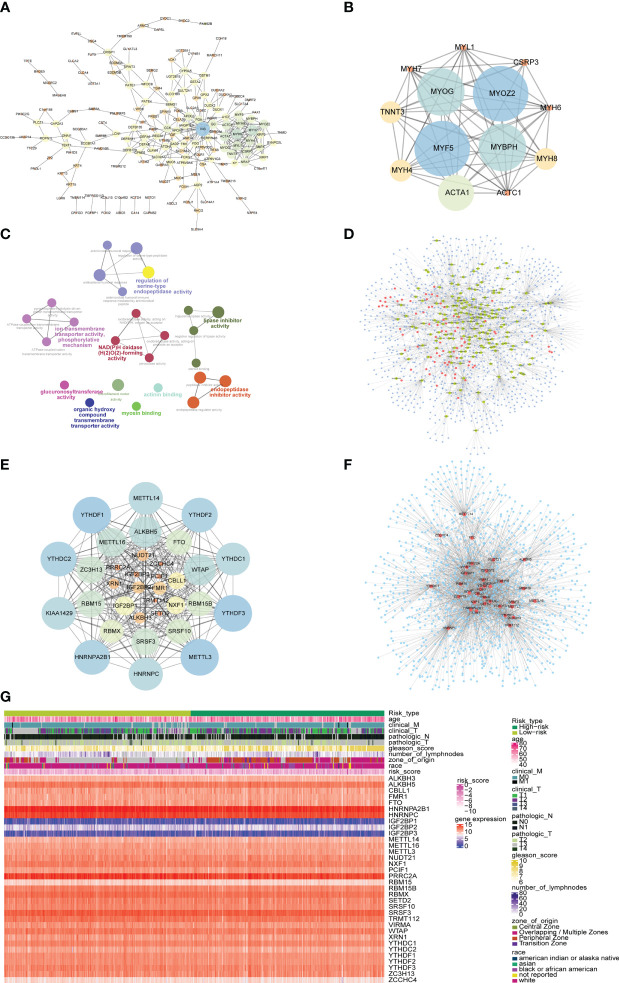
PPI network and mRNA-miRNA-lncRNA network of differentially expressed genes. **(A)** PPI network of differentially expressed genes. The node size represents the degree of the node. **(B)** The first subnet in the PPI network of differentially expressed gene. The node size represents the score of mcode. **(C)** Graph of enrichment analysis of PPI network of differentially expressed gene. **(D)** mRNA-miRNA-lncRNA network of differentially expressed genes. Blue nodes represent miRNAs, red nodes represent differentially expressed lncRNAs, and yellow nodes represent differentially expressed mRNAs. **(E)** PPI network of m^6^A-related gene. The node size indicates the degree of the node. **(F)** mRNA-miRNA network of m^6^A-related gene. Blue nodes represent miRNAs, and red nodes represent m^6^A-related genes. **(G)** The heat map of m^6^A-related genes, risk scores combined with clinicopathological characteristics.

In order to explore the mechanism affecting the difference between high-risk and low-risk groups, the PPI network of differentially expressed genes in a high-risk group and low-risk group was obtained from the String database, which was visualized by cytoscape (**Figure 11A**). The network contained 170 genes, where INS was also closely linked with 32 differentially expressed genes, while both MYH6 and MYH7 were linked with 18 differentially expressed genes. The functional interaction subnet was extracted by MCODE (**Figure 11B**). The ACTA1, ACTC1, and MYH4 in the subnet were all linked to multiple DEGs in PPI. To verify the functions of genes in the PPI, ClueGO functional enrichment analysis was performed, which showed that genes in PPI were significantly enriched in biological functions including ion transmembrane transporter activity, phosphorylative, regulation of serine-type endopeptidase activity mechanism, endopeptidase inhibitor activity, and glucuronosyltransferase activity (**Figure 11C**).

The differentially expressed mRNA and differentially expressed lncRNA were used to construct the mRNA-miRNA network and lncRNA-miRNA network, respectively. The intersection of the miRNAs in the two networks was taken to obtain the mRNA-miRNA-lncRNA network associated with patients in the high-risk group and the low-risk group (**Figure 11D**). The network contained 17 mRNA-miRNA-lncRNA relationships, including 91 miRNAs.

At the same time, the PPI network between m^6^A-related genes was constructed (**Figure 11E**). The network contained 278 interaction relationships and 34 m^6^A-related genes, among which METTL3, YTHDF1, and YTHDF3 were the three nodes with the highest degree.

Similarly, the mRNA-miRNA network of m^6^A-related genes was constructed (**Figure 11F**), and the network contained 34 m^6^A-related genes and 1121 miRNAs. The top 5 m^6^A-related genes were IGF2BP1 regulated by 241 miRNAs, HNRNPA2B1 regulated by 207 miRNAs, YTHDF1 regulated by 155 miRNAs, PRRC2A regulated by 144 miRNAs, and YTHDF3 regulated by 143 miRNAs. The top 4 of miRNAs that controlled multiple m^6^A-related genes simultaneously were hsa-mir-1-3p controlling 24 m^6^A-related genes, hsa-let-7b-5p controlling 20 m^6^A-related genes, hsa-mir-124-3p controlling 19 m^6^A-related genes, and hsa-mir-16-5p controlling 17 m^6^A-related genes. Moreover, the heatmap of m6A-related genes, risk scores combined with clinicopathological characteristics was shown to further explore the relationship among risk scores, m6A-related genes and clinicopathological characteristics (**Figure 11G**).

### Differences in immune characteristics and drug sensitivity prediction of patients in high-risk group and low-risk group

**Figure 12:**
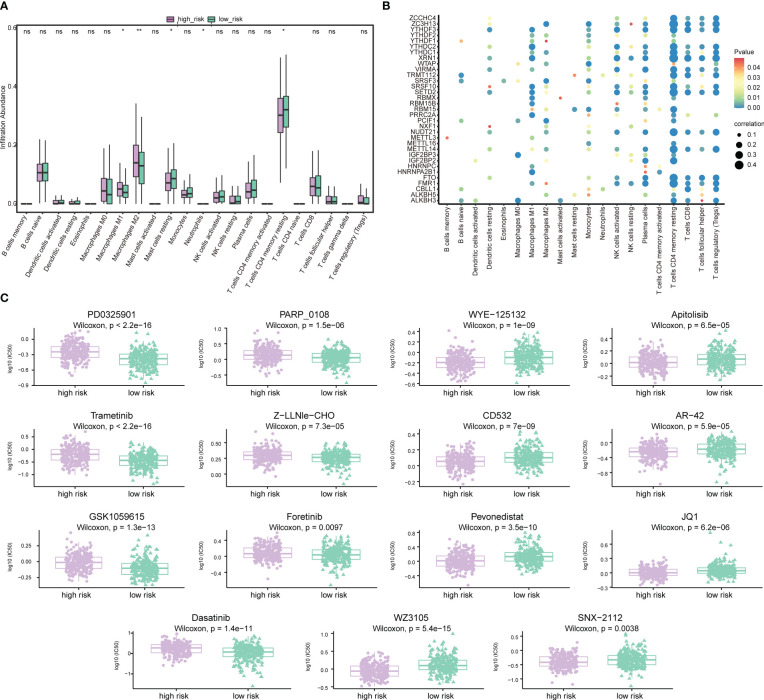
Association of risk score-m^6^A-related gene-immune cell infiltration and drug sensitivity. **(A)** Histogram of the level of immune cells infiltration between patients in a high-risk group and low-risk group. Light green represents the high-risk group, dark green represents the low-risk group, the horizontal axis represents immune cell subtypes, and the vertical axis represents the infiltration level of cells. **(B)** Correlation diagram between m^6^A-related genes and immune cells. The horizontal axis represents immune cell subtypes, the vertical axis represents m^6^A-related genes, the node size represents the absolute value of the correlation size, and the node color represents the significance level. **(C)** Differences in drug sensitivity between patients in the high-risk group and low-risk group. The horizontal axis indicates grouping, and the vertical axis indicates -log0 (IC50). *P < 0.05, **P < 0.01, ns, not significant.

Next, the effect of risk score on the overall immune profile and different infiltration levels of immune cell in PRAD patients was assessed, revealing that compared with the low-risk group, the levels of M1 macrophages and M2 macrophages in the high-risk group significantly increased, while the levels of mast cells resting and T cells CD4 memory resting significantly decreased (*P*< 0.05, **Figure 12A**). We further calculated the correlation between the level of immune cell and the expression level of m^6^A-related gene (**Figure 12B**), finding that resting memory CD4+ T cells and regulatory T cells (Tregs) were strongly correlated with multiple m^6^A-related genes (*P*< 0.05).

We also predicted the drug sensitivity of PRAD patients in the high-risk group and low-risk group, finding that patients in the low-risk group were more sensitive to PD0325901, trametinib, GSK1059615, dasatinib, PARP_0108 and Z-LLNle-CHO, while patients in the high-risk group were more sensitive to WZ3105, WYE-125132, CD532, pevonedistat, and other drugs (**Figure 12C**).

**Figure 13:**
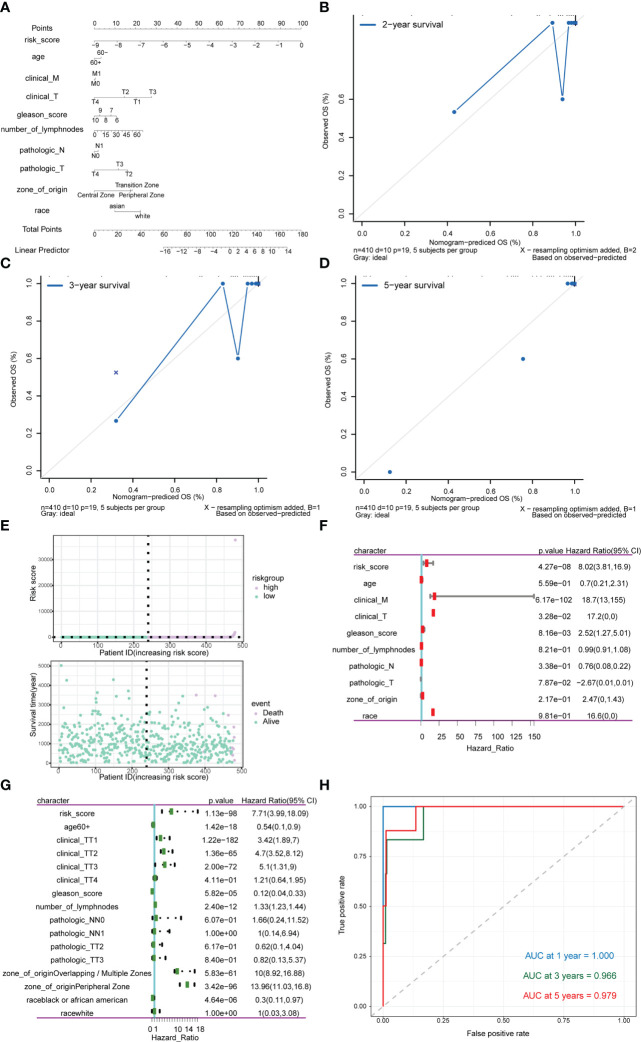
Analysis of the predictive power of risk scores for prognosis in PRAD patients. **(A–D)** Calibration curves of the nomogram. The horizontal axis is the survival predicted by the nomogram, and the vertical axis is the actual survival with repeated 1000 times each time. The curve shows the model had good predictive value of prognosis of patients for 2 years, 3 years and 5 years. **(E)** The risk group of the risk model. The horizontal axis shows the order of patient risk gradually increasing; the purple nodes represent patients with high-risk, the green nodes represent patients with low-risk, the vertical axis of the upper graph indicates the patient’s transformed risk score, and the vertical axis of the lower graph indicate survival time of patients. **(F)** HR and P values for risk scores by Univariate Cox regression analysis combined with clinicopathological features. **(G)** Multivariate Cox regression analysis of risk score combined with HR and P values of clinicopathological characteristics. The analysis showed that score of m^6^A group was an independent risk factor for the prognosis of PRAD patients. **(H)** Time-ROC curve of nomogram model for predicting 1-year survival, 3-year survival and 5-year survival of PRAD patients.

Subsequently, risk scores were combined with different clinicopathological characteristics to construct a predictive nomogram to predict OS in PRAD patients (**Figure 13A**). Moreover, the calibration curves showed good agreement between the 2-, 3-, and 5-year OS estimates by comparing the nomogram and actual value of OS (**Figures 13B–D**). We also assessed the effect of risk scores on the prognosis of PRAD patients. Dot plot of risk score showed that all death samples belonged to the high-risk group, and as the risk score increased, while the survival time of the patients was shorter (**Figure 13E**). Univariate and multivariate Cox analysis revealed that risk score was an independent risk factor for predicting the prognosis of PRAD patients (**Figures 13F, G**; **Supplementary Table 6**). By analyzing the correlation between m^6^A-related genes and risk scores or clinicopathological characteristics, it was found that the patients in the high-risk group were more in the middle and late stages. Patients in the high-risk group were older, and the cancerous sites were mostly in the central area with multiple points. m^6^A-related genes were significantly differentially expressed between patients in high-risk group and the low-risk group (**Figure 13G**). Besides, the time-ROC also showed that the predictive performance of the prognostic model was 100% for one-year survival, 96.9% for three-year survival, and 97.9% for five-year survival (**Figure 13H**).

### Expression validation of m^6^A-related gene in PCa cells

**Figure 14:**
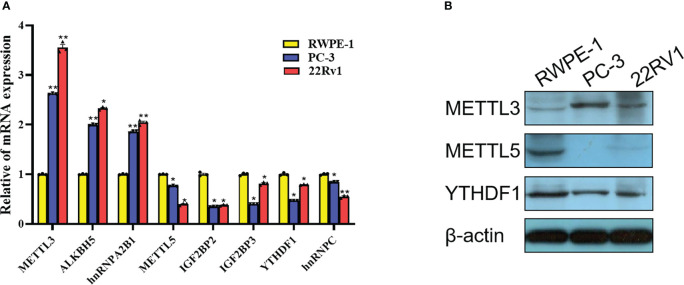
Expression validation of m^6^A-related gene in PCa cells. **(A)** Differences in mRNA expression of 8 m^6^A-related genes in 22Rv1, PC3 and RWPE-1 by RT-qPCR. **(B)** Differences in protein expression of METTL3, METTL5 and YTHDF1 in 22Rv1, PC3 and RWPE-1 by western blot. *P<0.05, **P<0.01.

Based on the comprehensive analysis of TCGA data and GEO data above, significant differences were found in expression of multiple m^6^A-related genes between PCa tissues and normal tissues, which were further verified at the cellular level. By comparing the expression of m^6^A-related genes in prostate normal cell line (RWPE-1) and 2 PCa cell lines (22Rv1 and PC3), 8 significantly DEGs were screened out by RT-qPCR, among which METTL3, ALKBH5 and hnRNPA2B1 were highly expressed in PCa cells, while METTL5, YTHDF1, IGF2BP2, IGF2BP3 and hnRNPC were lowly expressed in PCa cells (**Figure 14A**). Moreover, three m^6^A-related genes with the same expression trend as RT-qPCR results were screened out by Western blot, including METTL3, METTL5 and YTHDF1 (**Figure 14B**).

## Discussion

Cumulative evidence over the two decades suggested that various types of RNA modifications, such as 5-methylcytosine (m^5^C), m^6^A, inosine (I), and 2′-O-methylation (2′-O-Me) are implicated in PCa (6, 36–38). Among them, m^6^A has attracted the most attention due to the wide distribution of this modification across the human transcriptome. Yet, the interplay between m^6^A and PCa development is still not clearly understood. In this study, we systematically examined the relationship between expression of m^6^A regulators and progression/prognosis of PCa with the help of multiple bioinformatic tools. In addition, expression patterns of three candidates, i.e., METTL3, METTL5 and YTHDF1, have been successfully validated by experimental approaches.

As an important enzyme catalyzing the formation of m^6^A, METTL3 forms an m^6^A methyltransferase complex with METTL14, WTAP, and VIRMA to confer m^6^A marks to its binding RNA transcripts (39). One study revealed that METTL3 inhibits apoptosis of PCa cells *via* Sonic Hedgehog (SHH)-GLI pathway, indicating an oncogenic role of METTL3 during PCa progression (40). Another study demonstrated that METTL3 regulates the expression of Integrin β1 (ITGB1) through m^6^A-HuR-dependent mechanism, which subsequently promotes the bone metastasis of PCa (41). Notably, MYC, a well-known oncogene in PCa, was recently identified as a functional target of METTL3-mediated m^6^A modification. As a result, over-expression of MYC was sufficient to rescue the inhibitory effect of METTL3 knockdown on the tumorigenic activities of PCa cells (42). Consistent with these previous studies, we re-confirmed the elevated expression of METTL3 in PCa cells, identifying it as the key node of the PPI network and further unveiling its potential in the prognosis of advanced PCa.

Other than METTL3, which is responsible for more than 100,000 methylation events in humans, methyltransferase of METTL5 can only catalyze m^6^A in human 18S rRNA at position A1832 site, thus participating in translational control (43). Dysregulation of METTL5 has been revealed in breast cancer, pancreatic cancer and gastric cancer (44–46). To the best of our knowledge, this is the first study that reported METTL5 being downregulated in PCa samples compared to normal control. Considering the fact that METTL5 is mostly found to be upregulated in other cancer types and gas oncogenic functions, it will be interesting to investigate the reason for the downregulation of METTL5 in PCa and uncover its clinical relevance.

As an m^6^A reader, YTHDF1 interacts with several translation initiation factors to mediate the translation of m^6^A-modified transcripts (47). A recent study suggested that YTHDF1 is highly expressed in both PCa tissues and promotes the proliferation of PCa cells by regulating TRIM44 (48). Surprisingly, although we also identified YTHDF1 as a key node of both PPI and mRNA-miRNA networks, both RT-qPCR and western blot results showed a significant decrease of YTHDF1 in PCa cells compared to normal RWPE-1 cell line. This discrepancy may reflect the complexity of m^6^A-related regulation in PCa, which should be further investigated.

Increasing studies have revealed the m^6^A regulatory patterns of PCa and correlated these modification patterns with the tumor immune cell infiltration microenvironment characteristics (49–51). In addition, a recent paper found that m^6^A reader HNRNPC can regulate Treg cell abundance as a possible mechanism for m^6^A methylation-mediated response against CTLA-4, indicating that activation of the immune microenvironment by targeting m^6^A regulators may serve as a potential therapeutic approach for advanced PCa(52).Our study synthetically analyzed the relationship between the expression of m^6^A regulators and immune characteristics and drug sensitivity of PCa patients. In accordance with the previous reports, we confirmed that resting memory CD4+ T cells and Tregs are highly correlated with m^6^A-related genes (53, 54), while both high- and low-risk groups are sensitive to a number of therapeutic drugs. Some of these drugs are known to be effective in the treatment of PCa and have even been approved for clinical use (55–57). Thus, it will be informative to determine whether combinational treatment of m^6^A inhibitors and conventional PCa drugs could achieve a synergistic effect.

In the current study, four miRNAs, including hsa-mir-1-3p, hsa-let-7b-5p, hsa-mir-124-3p, and hsa-mir-16-5p were ranked as the top miRNAs, which dedicate the expression of m^6^A regulators. As expected, most of them have been validated to be closely associated with PCa progression and metastasis (58–61), which further confirmed our observations.

It still remains some limitations in this study. For instance, although the dysregulation m^6^A-related genes have been validated in a number of PCa cell lines, additional studies are needed to investigate the change of global m^6^A level in PCa specimen as compared with normal control. More importantly, the underlying mechanism by which the m^6^A modification is modulated in response to oncogenic signals during PCa development is yet to be discovered. Future efforts should be made to systematically deconstruct how the m^6^A-targeting axis promotes PCa tumorigenesis and unveil its clinical relevance.

## Conclusions

The present study systematically evaluated the expression pattern, functional network, and potential prognostic value of m^6^A regulators in PCa, which may provide novel insights into the understanding of PCa molecular pathology and facilitate the risk surveillance and clinical decision-making for patients diagnosed with PCa.

## Data availability statement

The original contributions presented in the study are included in the article/**Supplementary Material**. Further inquiries can be directed to the corresponding author.

## Author contributions

CL: Conception/design, collection and/or assembly of data, data analysis and interpretation, and manuscript writing. DP: Provision of study material, collection and/or assembly of data, and manuscript writing. YG, LZ, WH, BW, PY, and WX: Provision of study material and collection and/or assembly of data. LW: Conception/design and final approval of manuscript. All authors contributed to the article and approved the submitted version.
