# Supplementary material for: The m6A methylation landscape, molecular characterization and clinical relevance in prostate adenocarcinoma
Source: Front Immunol. 2023 Mar 23;14:1086907. doi: 10.3389/fimmu.2023.1086907 (PMC10076583; doi:10.3389/fimmu.2023.1086907)
Supplement: Supplementary file 1 [file Table_1.docx]

**Table S1.** Primers used in this study

| **Primer Name** | **Forward primer** | **Reverse Primer** |
| --- | --- | --- |
| METTL3 | TTGTCTCCAACCTTCCGTAGT | CCAGATCAGAGAGGTGGTGTAG |
| METTL5 | AGAGTCGCCTGCAACAAGTG | AATCCAACACACAACCCTGCT |
| METTL14 | AGTGCCGACAGCATTGGTG | GGAGCAGAGGTATCATAGGAAGC |
| METTL16 | ACGAGGCTAGATGGCTTCAC | ATTAAGGCTCACTCTTCCATTCAG |
| WTAP | CTGCCTGGAGAGGATTCAAG | AAGATCTGTGTACTTGCCCTCC |
| ZC3H13 | ATAGCACATCCCGAAGACCC | TTACGGCACTGTGTCTCTGC |
| HAKAI | CGAATCATGGATCACACTGACAAT | CTTCATCACCAGGTGGAGCC |
| KIAA1429 | CGAGCGCTGAGCAAAGTTC | GGAGATGTCTCTCCATATGCTC |
| RBM15 | ACGACCCGCAACAATGAAG | GGAAGTCGAGTCCTCACCAC |
| RBM15B | ATCTGAGGTGGAGCTGCGAA | AGAGACGAGTGGTGGGGTTG |
| FTO | GCTGCTTATTTCGGGACCTG | AGCCTGGATTACCAATGAGGA |
| ALKBH5 | CGGCGAAGGCTACACTTACG | CCACCAGCTTTTGGATCACCA |
| YTHDF1 | GAGGTGGTGCGCAAGGAAC | ACACACTGGAGCTGACCAAG |
| YTHDF2 | CTCTTGGAGCAGTACAAAATGGA | GGAGGGACTGTAGTAACTGGG |
| YTHDF3 | TTCAGTACAAAACGGTTCGATTCA | ATTGGTGGATAGCTGTTACTCTGA |
| YTHDC1 | AGAGTCATATGCAGATCAAACCAGT | AGGAGATCCTCCGTGATGTGA |
| YTHDC2 | AAAAGTAAAGGATTCCTCAGTTCCT | GCAACTCTTTCAGCCACAGC |
| IGF2BP1 | GGCAGGCTGACGAGGTTCC | GGTAAGGTCTTGCAACGAGGA |
| IGF2BP2 | AGCCTGTCACCATCCATGC | CTTCGGCTAGTTTGGTCTCATC |
| IGF2BP3 | CAGCTCCAGGAAATGCTAGTG | GTCAACTTTGTAGCGCTGGC |
| eIF3 | TGCGACTTGCAGGTTCGTAT | TGCAGTGACAGCCAACTGAT |
| HNRNPC | CTCCCCTTCTTGTTTTCGGC | TGCCATCCTCTCCTGCTACA |
| HNRNPG | ATGGTGGAAGCAGTCGCTAT | CCCGATCACGACCACTTGA |
| HNRNPA2B1 | TCCTTTGGAGAGGAAAAAGAGAGA | GCAGGATCCCTCATTACCACA |
| H-β-actin | TTCCTTCCTGGGCATGGAGTC | TCTTCATTGTGCTGGGTGCC |
