# Supplementary material for: The m6A methylation landscape, molecular characterization and clinical relevance in prostate adenocarcinoma
Source: Front Immunol. 2023 Mar 23;14:1086907. doi: 10.3389/fimmu.2023.1086907 (PMC10076583; doi:10.3389/fimmu.2023.1086907)
Supplement: Supplementary file 2 [file Table_2.docx]

**Table S2.** Antibodies used in this study

| **Antibody Name** | **Source** | **Catalog number** | **Company** | **Dilution** |
| --- | --- | --- | --- | --- |
| METTL3 | Rabbit | A8370 | Abclonal | 1：1000 |
| METTL5 | Rabbit | YN4415 | Immunoway | 1：1000 |
| ALKBH5 | Rabbit | YT6218 | Immunoway | 1：1000 |
| YTHDF1 | Rabbit | YT1283 | Immunoway | 1：1000 |
| IGF2BP2 | Rabbit | YT2284 | Immunoway | 1：1000 |
| RNPC1/2 | Rabbit | YT2194 | Immunoway | 1：1000 |
| RNPA2/B1 | Rabbit | YT2193 | Immunoway | 1：1000 |
| β-actin | Mouse | YM3028 | Immunoway | 1：10000 |
