# Supplementary material for: The m6A methylation landscape, molecular characterization and clinical relevance in prostate adenocarcinoma
Source: Front Immunol. 2023 Mar 23;14:1086907. doi: 10.3389/fimmu.2023.1086907 (PMC10076583; doi:10.3389/fimmu.2023.1086907)
Supplement: Supplementary file 3 [file Table_3.docx]

**Supplementary Table 3**. Baseline of TCGA-PRAD patients

|  | low-risk (236) | high-risk (245) |
| --- | --- | --- |
| age-60+ | 131 | 134 |
| clinical_T-T1 | 81 | 94 |
| clinical_T-T2 | 82 | 88 |
| clinical_T-T3 | 20 | 30 |
| clinical_T-T4 | 2 | 0 |
| gleason_score->7 | 93 | 103 |
| number_of_lymphnodes->10 | 87 | 92 |
| pathologic_N-N0 | 160 | 177 |
| pathologic_N-N1 | 38 | 37 |
| pathologic_T-T2 | 94 | 92 |
| pathologic_T-T3 | 133 | 145 |
| zone_of_origin- Multiple Zones | 65 | 53 |
| zone_of_origin-Peripheral Zone | 60 | 76 |
| race-black or african american | 25 | 31 |
| race-white | 200 | 198 |
