# Supplementary material for: The m6A methylation landscape, molecular characterization and clinical relevance in prostate adenocarcinoma
Source: Front Immunol. 2023 Mar 23;14:1086907. doi: 10.3389/fimmu.2023.1086907 (PMC10076583; doi:10.3389/fimmu.2023.1086907)
Supplement: Supplementary file 4 [file Table_4.docx]

**Supplementary Table 4.** GO enrichment analysis of differentially expressed genes

| **Category** | **ID** | **Description** | **P value** |
| --- | --- | --- | --- |
| BP | GO:0030049 | muscle filament sliding | 7.53046E-09 |
| BP | GO:0033275 | actin-myosin filament sliding | 7.53046E-09 |
| BP | GO:0055002 | striated muscle cell development | 1.72924E-07 |
| BP | GO:0030239 | myofibril assembly | 2.25549E-07 |
| BP | GO:0042403 | thyroid hormone metabolic process | 2.85834E-07 |
| BP | GO:0010927 | cellular component assembly involved in morphogenesis | 1.38035E-06 |
| BP | GO:0006590 | thyroid hormone generation | 2.49033E-06 |
| BP | GO:0006575 | cellular modified amino acid metabolic process | 1.07603E-05 |
| BP | GO:0003341 | cilium movement | 1.21455E-05 |
| BP | GO:0048240 | sperm capacitation | 1.35458E-05 |
| MF | GO:0055102 | lipase inhibitor activity | 4.02754E-05 |
| MF | GO:0004866 | endopeptidase inhibitor activity | 0.000189787 |
| MF | GO:0030414 | peptidase inhibitor activity | 0.000252243 |
| MF | GO:0000146 | microfilament motor activity | 0.000284045 |
| MF | GO:0061135 | endopeptidase regulator activity | 0.000306584 |
| MF | GO:0004857 | enzyme inhibitor activity | 0.000391953 |
| MF | GO:0015078 | proton transmembrane transporter activity | 0.000477695 |
| MF | GO:0061134 | peptidase regulator activity | 0.001120914 |
| MF | GO:0008307 | structural constituent of muscle | 0.001195847 |
| CC | GO:0030017 | sarcomere | 7.30771E-09 |
| CC | GO:0030016 | myofibril | 2.75029E-08 |
| CC | GO:0043292 | contractile fiber | 4.1403E-08 |
| CC | GO:0005859 | muscle myosin complex | 4.15063E-07 |
| CC | GO:0016460 | myosin II complex | 1.5507E-06 |
| CC | GO:0032982 | myosin filament | 3.419E-06 |
| CC | GO:0031674 | I band | 1.72643E-05 |
| CC | GO:0016324 | apical plasma membrane | 3.51035E-05 |
| CC | GO:0045177 | apical part of cell | 5.54307E-05 |
