# Supplementary material for: The m6A methylation landscape, molecular characterization and clinical relevance in prostate adenocarcinoma
Source: Front Immunol. 2023 Mar 23;14:1086907. doi: 10.3389/fimmu.2023.1086907 (PMC10076583; doi:10.3389/fimmu.2023.1086907)
Supplement: Supplementary file 5 [file Table_5.docx]

**Supplementary Table 5.** KEGG pathway enrichment analysis of differentially expressed genes

| **Category** | **ID** | **Description** | **P value** |
| --- | --- | --- | --- |
| KEGG_PATHWAY | hsa04918 | Thyroid hormone synthesis | 6.31638E-06 |
| KEGG_PATHWAY | hsa05204 | Chemical carcinogenesis - DNA adducts | 4.53137E-05 |
| KEGG_PATHWAY | hsa04972 | Pancreatic secretion | 4.78794E-05 |
| KEGG_PATHWAY | hsa00982 | Drug metabolism - cytochrome P450 | 5.77404E-05 |
| KEGG_PATHWAY | hsa00980 | Metabolism of xenobiotics by cytochrome P450 | 9.07352E-05 |
| KEGG_PATHWAY | hsa00830 | Retinol metabolism | 0.000446141 |
| KEGG_PATHWAY | hsa04721 | Synaptic vesicle cycle | 0.000839229 |
| KEGG_PATHWAY | hsa04260 | Cardiac muscle contraction | 0.001374565 |
| KEGG_PATHWAY | hsa04976 | Bile secretion | 0.001521534 |
| KEGG_PATHWAY | hsa04950 | Maturity onset diabetes of the young | 0.00183816 |
