# Supplementary material for: The m6A methylation landscape, molecular characterization and clinical relevance in prostate adenocarcinoma
Source: Front Immunol. 2023 Mar 23;14:1086907. doi: 10.3389/fimmu.2023.1086907 (PMC10076583; doi:10.3389/fimmu.2023.1086907)
Supplement: Supplementary file 6 [file Table_6.docx]

**Supplementary Table 6.** GSEA analysis between the high-risk group and low-risk group

| ID | ES | NES | P value |
| --- | --- | --- | --- |
| go_myofibril_assembly | 0.77755 | 2.294192 | 1E-10 |
| go_contractile_fiber | 0.678063 | 2.239274 | 1E-10 |
| go_i_band | 0.688563 | 2.201593 | 1E-10 |
| go_cellular_component_assembly_involved_in_morphogenesis | 0.693215 | 2.182637 | 1E-10 |
| go_muscle_cell_development | 0.619221 | 2.013047 | 1E-10 |
| go_muscle_contraction | 0.598394 | 1.999042 | 1E-10 |
| go_muscle_system_process | 0.584811 | 1.958343 | 1E-10 |
| go_striated_muscle_cell_differentiation | 0.572605 | 1.905171 | 1E-10 |
| go_muscle_cell_differentiation | 0.559929 | 1.869941 | 1E-10 |
| go_muscle_tissue_development | 0.526487 | 1.761725 | 1E-10 |
| go_muscle_organ_development | 0.522538 | 1.748589 | 1E-10 |
| go_nucleosome_organization | -0.47918 | -2.08126 | 1E-10 |
| go_regulation_of_gene_expression_epigenetic | -0.4523 | -2.08633 | 1E-10 |
| go_chromatin_assembly_or_disassembly | -0.48176 | -2.14337 | 1E-10 |
| go_telomere_organization | -0.49147 | -2.19533 | 1E-10 |
| go_dna_packaging | -0.47216 | -2.23563 | 1E-10 |
| go_condensed_chromosome | -0.49422 | -2.25301 | 1E-10 |
| go_dna_dependent_dna_replication | -0.52318 | -2.28824 | 1E-10 |
| go_nucleosome_assembly | -0.54974 | -2.39993 | 1E-10 |
| go_regulation_of_chromosome_segregation | -0.58712 | -2.42233 | 1E-10 |
| kegg_cell_cycle | -0.52388 | -2.2444877 | 1.20E-09 |
| kegg_cytokine_cytokine_receptor_interaction | 0.52651 | 1.7264938 | 5.88E-09 |
| kegg_hypertrophic_cardiomyopathy_hcm | 0.65529 | 1.9810436 | 2.20E-08 |
| kegg_dilated_cardiomyopathy | 0.63471 | 1.9321012 | 1.12E-07 |
| kegg_ribosome | -0.53818 | -2.1202682 | 2.64E-07 |
| kegg_spliceosome | -0.45281 | -1.936332 | 5.6E-07 |
| kegg_calcium_signaling_pathway | 0.528468 | 1.694647 | 2.79E-06 |
| kegg_arrhythmogenic_right_ventricular_cardiomyopathy_arvc | 0.612945 | 1.8310956 | 1.16E-05 |
| kegg_neuroactive_ligand_receptor_interaction | 0.47033 | 1.5410205 | 1.82E-05 |
| kegg_systemic_lupus_erythematosus | -0.41410 | -1.7716184 | 1.97E-05 |
