# Supplementary material for: The m6A methylation landscape, molecular characterization and clinical relevance in prostate adenocarcinoma
Source: Front Immunol. 2023 Mar 23;14:1086907. doi: 10.3389/fimmu.2023.1086907 (PMC10076583; doi:10.3389/fimmu.2023.1086907)
Supplement: Supplementary file 7 [file Table_7.docx]

**Supplementary Table 7.** Univariate and multivariate Cox analysis of OS prediction in PRAD patients based on risk model

| character | P value | HR | HR 95% lower | HR 95% upper |
| --- | --- | --- | --- | --- |
| risk_score | 4.27E-08 | 8.02 | 3.81 | 16.9 |
| age | 0.559 | 0.701 | 0.213 | 2.31 |
| clinical_M | 6.17E-102 | 18.7 | 3 | 155 |
| clinical_T | 0.0328 | 17.2 | 0 | 0 |
| gleason_score | 0.00816 | 2.52 | 1.27 | 5.01 |
| number_of_lymphnodes | 0.821 | 0.991 | 0.913 | 1.08 |
| pathologic_N | 0.338 | 0.755 | 0.0824 | 0.219 |
| pathologic_T | 0.0787 | -2.67 | 0.0051 | 0.0081 |
| zone_of_origin | 0.217 | 2.47 | 0 | 1.43 |
| race | 0.981 | 16.6 | 0 | 0 |

| character | P value | HR | HR 95% lower | HR 95% upper |
| --- | --- | --- | --- | --- |
| risk_score | 1.13E-98 | 7.71 | 3.988763 | 18.08843 |
| age60+ | 1.42E-18 | 0.543498 | 0.101788 | 0.902009 |
| clinical_TT1 | 1.23E-182 | 3.42 | 1.89 | 7.001 |
| clinical_TT2 | 1.37E-65 | 4.7 | 3.518879 | 8.116484 |
| clinical_TT3 | 2.01E-72 | 5.1 | 1.31 | 31.91 |
| clinical_TT4 | 0.411861 | 1.21 | 6.41E-01 | 1.95 |
| gleason_score | 5.82E-05 | 0.117 | 0.041314 | 0.333641 |
| number_of_lymphnodes | 2.40E-12 | 1.327472 | 1.226374 | 1.436903 |
| pathologic_NN0 | 0.607904 | 1.660301 | 0.239362 | 11.51647 |
| pathologic_NN1 | 1 | 1 | 0.144168 | 6.936372 |
